# Excimer light 308 nm therapy for alopecia areata that is resistant to other treatments: a clinical study of three patients^☆^

**DOI:** 10.1016/j.abd.2026.501377

**Published:** 2026-06-16

**Authors:** Daniela Morales Hermosilla, Josefa Catalán Lobo, Rocío González Cobos, Silvia Guerrero Cornejo

**Affiliations:** a Department of Dermatology, University of Chile, Santiago, Chile; b Department of Medicine, Public Assistance Emergency Hospital, Santiago, Chile

Dear Editor,

Alopecia areata (AA) is an autoimmune, T-cell-mediated disease that causes hair loss on the scalp and/or other areas of the body.^1^, ^2^ It is the second most common cause of non-scarring alopecia and significantly affects patients’ quality of life.^1^, ^2^, ^3^, ^4^, ^5^, ^6^

The cornerstone of treatment has traditionally been topical, intralesional, or systemic corticosteroid therapy, although responses are unpredictable.^2^, ^5^, ^6^ Other alternatives include expectant management, immunotherapies, or systemic immunosuppressants.^1^, ^2^, ^3^, ^6^ Additional therapeutic options exist, such as JAK inhibitors and photobiomodulation-based therapies, including excimer light, a subtype of Narrow-Band-UVB (NB-UVB) phototherapy that emits a monochromatic and coherent wavelength.^5^, ^6^ Unlike conventional NB-UVB phototherapy, it provides deeper penetration and a more localized effect, enabling targeted treatment of difficult-to-reach areas. This translates into fewer sessions, shorter treatment times, and lower cumulative UVB dosage, thereby reducing the risk of adverse effects.^7^, ^8^ It is considered safe and does not induce systemic adverse effects, with only local events reported, such as erythema, scaling, pruritus, localized hyperpigmentation, and mild pain.^1^, ^6^, ^9^

Its effectiveness has not been demonstrated in cases of alopecia universalis or totalis,^7^, ^8^ and there is no consensus regarding the most effective protocol for the use of this device or which patients benefit most.^5^

We present three patients with AA refractory to conventional therapy, considering the potential of excimer light as an effective and safe therapeutic option. Treatment sessions were administered with variable frequency and dosage parameters depending on tolerance and clinical response.

## Case 1

**Figure 1:**
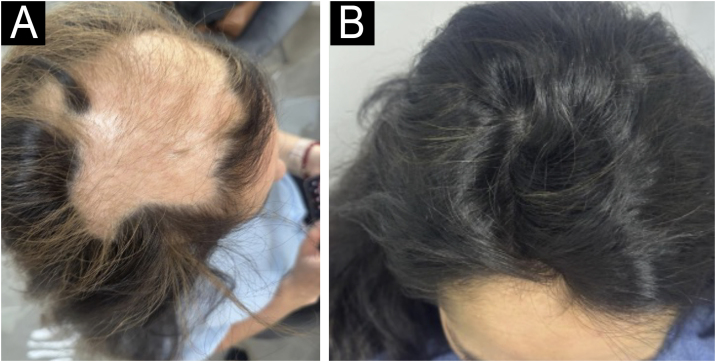
(A) Case 1. Photograph of the patient before the start of excimer laser therapy. An alopecic patch is observed affecting the biparietal and bitemporal coronal areas of the scalp. The SALT score was 54. (B) Same patient, photograph after 23 excimer laser sessions and at the 3-month follow-up after discontinuation of therapy. Hair regrowth is evident, and the SALT score is 0.

A 43-year-old woman, diagnosed with AA during the sixth month postpartum, presented with an extensive alopecic patch affecting the coronal, biparietal, and bitemporal regions, with a SALT score of 54 (Fig. 1A). Trichoscopy revealed yellow dots and fine long hairs. After one and a half years of treatment with methotrexate, deflazacort, topical clobetasol, and oral minoxidil, and due to only a partial therapeutic response, 308 nm excimer light was added. She underwent 23 twice-weekly sessions with escalating doses up to 800 mJ, with no adverse effects. She achieved an excellent response, SALT 0, with trichoscopy showing no signs of activity. No recurrence was observed after 6-months of follow-up (Fig. 1B).

## Case 2

**Figure 2:**
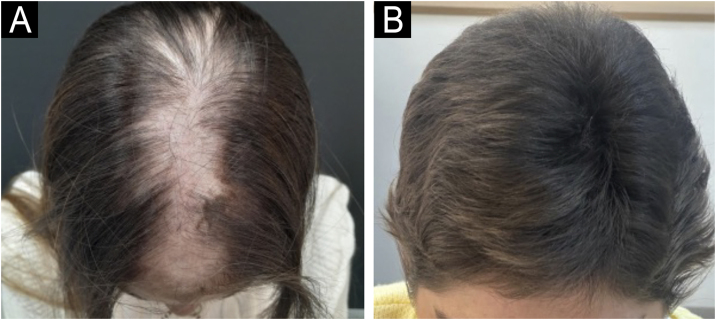
(A) Case 2. Photograph of a patient diagnosed with alopecia areata with a large alopecic patch in the frontal area, midline, parietal, temporal and occipital areas of the scalp, SALT index 46. (B) Same patient, after one month of discontinuation of therapy, after having received 16 sessions of excimer light with SALT 0.

A 30-year-old woman with no medical history presented with four months of burning sensation and pruritus of the scalp. A single frontal alopecic plaque was noted; despite intralesional and topical corticosteroids, the alopecia progressed throughout the scalp. The SALT score was calculated at 46 (Fig. 2A). Trichoscopy showed yellow dots, black dots, broken hairs, coudability hairs, and exclamation-mark hairs, consistent with active disease. Histopathology supported the diagnosis of alopecia areata. After four months of treatment with intralesional corticosteroids, clobetasol 0.05%, and topical tacrolimus 0.1% with poor response, intralesional corticosteroids were discontinued, and deflazacort plus excimer light were initiated. After 16 weekly sessions with escalating doses up to 250 mJ, she achieved a SALT score of 0 (Fig. 2B). No adverse effects occurred. She is currently receiving twice-weekly oral corticosteroids and oral minoxidil, without relapse after three months of follow-up.

## Case 3

**Figure 3:**
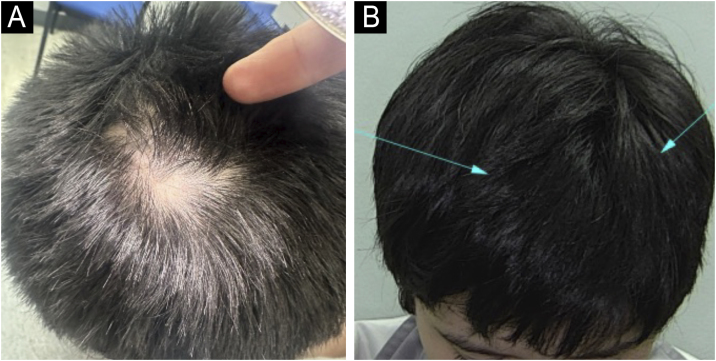
(A) Case 3. Photograph of a pediatric patient before starting 308 nm excimer laser treatment. Refractory alopecia patches are observed on the vertex and occipital region. (B) After 16 sessions of 308 nm excimer laser treatment, significant hair regrowth is observed (light blue arrows).

A 12-year-old boy with no medical history and a diagnosis of alopecia totalis was treated with diphencyprone, clobetasol 0.05%, oral deflazacort, and topical minoxidil 5%. He showed a partial response after three years of treatment, with persistent alopecic areas with signs of activity on the occipital, parietal, and temporal regions. Trichoscopy revealed black dots, broken hairs, and exclamation-mark hairs. Due to poor tolerance to intralesional corticosteroids secondary to pain, excimer light was added to the refractory alopecic patches (Fig. 3A). After 16 twice-weekly sessions with escalating doses up to 700 mJ, he showed hair regrowth (Fig. 3B). Although vellus and pigtail hairs were observed, trichoscopy revealed no signs of activity. He experienced erythema lasting longer than 48 hours on two occasions

AA poses a therapeutic challenge due to its variable and unpredictable responses.^2^, ^6^ However, excimer light demonstrates a favorable safety profile, good tolerability, and absence of systemic adverse effects.^1^, ^6^ In all three cases presented, hair regrowth was observed, assessed by SALT score and trichoscopy, with minimal adverse events.

This therapy appears to be a promising and safe therapeutic alternative for patients with refractory AA, complementary to other strategies. Given the lack of consensus regarding standardized protocols, additional studies are needed to further define its therapeutic role and explore its potential use as monotherapy.

## ORCID IDs

Daniela Morales Hermosilla: 0009-0000-7753-3372

Josefa Catalán Lobo: 0009-0002-2431-1199

Silvia Guerrero Cornejo: 0009-0005-1925-3854

## Research data availability

Does not apply.

## Financial support

None declared.

## Authors' contributions

All authors contributed to the completion of this article.

Daniela Morales H: The study concept and design; Data collection.

Josefa Catalán L: Effective participation in the research guidance.

Rocío González C: Writing of the manuscript.

Silvia Guerrero C: Final approval of the final version of the manuscript.

## Conflicts of interest

None declared.
